# Detection of Children/Youth With Fetal Alcohol Spectrum Disorder Through Eye Movement, Psychometric, and Neuroimaging Data

**DOI:** 10.3389/fneur.2019.00080

**Published:** 2019-02-18

**Authors:** Chen Zhang, Angelina Paolozza, Po-He Tseng, James N. Reynolds, Douglas P. Munoz, Laurent Itti

**Affiliations:** ^1^Neuroscience Graduate Program, University of Southern California, Los Angeles, CA, United States; ^2^Center for Neuroscience Studies, Queen's University, Kingston, ON, Canada; ^3^Department of Neurobiology, Duke University, Durham, NC, United States; ^4^Center for Neuroengineering, Duke University, Durham, NC, United States; ^5^Department of Biomedical and Molecular Science, Queen's University, Kingston, ON, Canada; ^6^Department of Computer Science, University of Southern California, Los Angeles, CA, United States

**Keywords:** fetal alcohol spectrum disorder (FASD), eye movements, psychometrics, DTI, early screening

## Abstract

**Background:** Fetal alcohol spectrum disorders (FASD) is one of the most common causes of developmental disabilities and neurobehavioral deficits. Despite the high-prevalence of FASD, the current diagnostic process is challenging and time- and money- consuming, with underreported profiles of the neurocognitive and neurobehavioral impairments because of limited clinical capacity. We assessed children/youth with FASD from a multimodal perspective and developed a high-performing, low-cost screening protocol using a machine learning framework.

**Methods and Findings:** Participants with FASD and age-matched typically developing controls completed up to six assessments, including saccadic eye movement tasks (prosaccade, antisaccade, and memory-guided saccade), free viewing of videos, psychometric tests, and neuroimaging of the corpus callosum. We comparatively investigated new machine learning methods applied to these data, toward the acquisition of a quantitative signature of the neurodevelopmental deficits, and the development of an objective, high-throughput screening tool to identify children/youth with FASD. Our method provides a comprehensive profile of distinct measures in domains including sensorimotor and visuospatial control, visual perception, attention, inhibition, working memory, academic functions, and brain structure. We also showed that a combination of four to six assessments yields the best FASD vs. control classification accuracy; however, this protocol is expensive and time consuming. We conducted a cost/benefit analysis of the six assessments and developed a high-performing, low-cost screening protocol based on a subset of eye movement and psychometric tests that approached the best result under a range of constraints (time, cost, participant age, required administration, and access to neuroimaging facility). Using insights from the theory of value of information, we proposed an optimal annual screening procedure for children at risk of FASD.

**Conclusions:** We developed a high-capacity, low-cost screening procedure under constrains, with high expected monetary benefit, substantial impact of the referral and diagnostic process, and expected maximized long-term benefits to the tested individuals and to society. This annual screening procedure for children/youth at risk of FASD can be easily and widely deployed for early identification, potentially leading to earlier intervention and treatment. This is crucial for neurodevelopmental disorders, to mitigate the severity of the disorder and/or frequency of secondary comorbidities.

## Introduction

Fetal alcohol spectrum disorder (FASD) is the most common preventable developmental disorder, resulting from prenatal alcohol exposure (1). The estimated prevalence of FASD among school-age children may be as high as 2–5% in the U.S. (2, 3), and over 1% in Canada (4). Costs associated with FASD, in areas such as health care, special education, and social services, can run into billions of dollars annually, which places a large burden on both families and society (5, 6).

Early diagnosis of FASD is important in that it can lead to early interventions that reduce the risk of developing secondary disabilities (7). Despite the high prevalence of FASD, the clinical diagnosis can be both challenging and time consuming. It currently requires a confirmed history of the prenatal alcohol exposure and a comprehensive profile of central nervous system and neurobehavioral deficits, which are often difficult to obtain. The high rate of co-morbidity with other developmental disorders such as attention deficit hyperactivity disorder (ADHD) (8) may also contribute to misdiagnosis. The diagnostic process may take up to two full days requiring a multidisciplinary team comprised of a physician, psychologist, facial dysmorphologist and occupational therapist, and the result can vary from clinic to clinic because of the wide spectrum of deficits (9). Therefore, an easy, objective, and effective procedure which can assess the deficits and differentiate the neurological groups is needed as a screening tool for children at risk for FASD. Most of the screening or discrimination studies, however, have so far relied on demographic, behavioral, physical, psychometric, face morphometric analysis, and history of maternal alcohol consumption (10–14), which are not time- and cost-efficient.

Multiple brain regions including the corpus callosum are affected in FASD (15). Studies using diffusion tensor imaging (DTI), a magnetic resonance imaging technique widely used to examine the structural integrity of white matter tracks, have revealed abnormalities in white matter tracts, such as altered fractional anisotropy (FA) and mean diffusivity (MD) within different parts of the corpus callosum (16). Moreover, these structural anomalies have been correlated with saccadic eye movement control and inhibition deficits in children with FASD (17, 18). Psychometric tests have also been used to assess the functional and cognitive deficits caused by prenatal alcohol exposure. Children with FASD received lower scores on attention tests, inhibitory control and performed poorer in working memory tests and visuospatial processing (19–21). They also showed difficulties in learning and language such as verbal information acquisition and word comprehension (22, 23). Saccadic eye movements are the rapid shifts that redirect the line of sight to foveate new visual targets (24). The execution and control of saccadic eye movements involve multiple cortical and subcortical brain areas, reflecting the automatic, executive, and cognitive functions of the individual (25–27). Children with FASD have significantly poorer saccade control with more variability, slower saccadic reaction times, and more timing and direction errors compared to typically developing controls (18, 20, 28). Biometric signatures decoded from eye tracking during free viewing of natural videos have been shown to help explain the deficits and to differentiate clinical populations for various neurodevelopmental and neurodegenerative disorders, such as FASD, ADHD, and Parkinson's disease (29, 30). Related work using static natural images has shown promising results in further understanding autism spectrum disorder (31).

In this paper, we propose a machine learning framework to address the various outcome measures that are used to quantify deficits across multiple domains in children/youth with FASD, and to use these measures to differentiate the FASD group from typically developing controls. We utilized data from eye movement behaviors, psychometric test scores, and DTI of the brain to construct a new, multimodal classifier that demonstrates high performance in identifying the clinical population. Considering time, cost, age restriction, required administration, and accessibility of different measurements, we also propose a high-throughput and low-cost screening procedure with high expected monetary benefit, based on eye movement recordings for children with FASD, which could be widely deployed and lead to the earlier intervention and treatment that is crucial for neurodevelopmental disorders.

To quantify the potential benefits of early screening on a large scale, we used tools from the theory of value of information (32). The expected value of information is computed under situations where a decision maker has to choose whether to spend some money to obtain an additional piece of information which may lead to a better decision and to long-term benefits. Here, we propose a cost-benefit model based on this theory to evaluate the expected benefits of our screening procedure.

## Materials and Methods

### Participants

The participants in this study were part of a large, multi-site investigation funded by the Kids Brain Health Network (formerly NeuroDevNet), a Network of Centers of Excellence in developmental neuroscience (33). Participants were recruited from five different communities in three provinces in Canada. This study included children/youth aged 5–18 years who were either typically developing healthy controls (*n* = 116) or had received a diagnosis of an FASD (*n* = 91) according to the Canadian Guidelines (9). Demographic information of the participants is summarized in Table 1. Each participant was tested in up to six different assessment procedures described below. A summary of the numbers of participants that completed each and all assessments are provided in Table 2.

**Table 1:** Demographic.

		**FASD**	**Control**
n (%)		91 (44.0)	116 (56.0)
Age mean ± std years (range)		11.9 ± 3.4 (5–18)	10.8 ± 3.5 (5–18)
Males n (%)		46 (50.5)	51 (44.0)
Right handed n (%)		69 (75.8)	85 (73.3)
Diagnostic subtype n (%)	ARND	62 (68.1)	–
	FAS	10 (11.0)	–
	pFAS	19 (20.9)	–

**Table 2:** Participants for different assessments.

	**Prosaccade**	**M-G saccade**	**Antisaccade**	**Natural viewing**	**DTI**	**Psychometric**	**ALL**
n(FASD)	71	61	67	47	41	58	22
n(Control)	115	93	106	53	35	71	24

All experimental procedures were reviewed and approved by the Human Research Ethics Board at Queen's University, the University of Manitoba, the University of Alberta, and the University of Southern California (USC). Written informed consent was obtained from a parent or legal guardian before the protocol was administered and children completed a written assent form before study participation.

### Structured Saccadic Eye Movement Tasks

Participants were comfortably seated on a stable chair in a quiet, darkened room. Eye position was recorded using the Eyelink 1,000 system (SR Research, Kanata, ON). A 17-inch LCD monitor with a built-in infrared illuminator and infrared camera was placed ~60 cm from the left eye. The coordinates of the left pupil were sampled in the vertical and horizontal axes at 500 Hz. Eye position was calibrated using 9 sequential visual targets positioned around the screen (8 around the periphery and one central). The participants were asked to fixate on each target when it flashed. After initial calibration, the process was repeated to validate that the average error between fixation and target was < 2° and that no loss of eye tracking occurred. The performance of each participant was assessed in three saccadic eye movement tasks: prosaccade (ProSac), antisaccade (AntiSac), and a memory guided saccade (MGSac) task.

**Figure 1:**
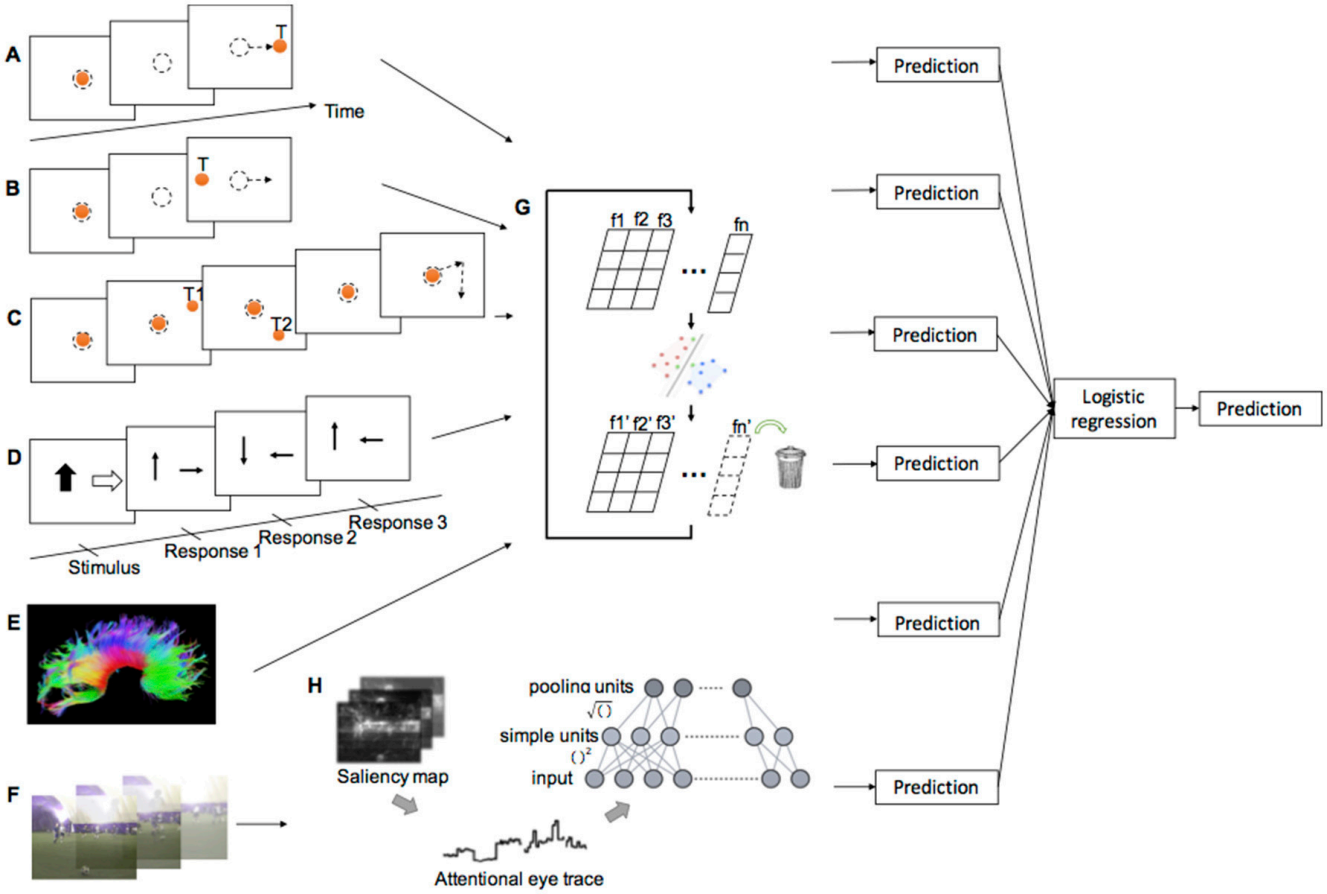
Illustration of experimental methods and classification procedure. **(A)** Prosaccade task. **(B)** Antisaccade task. **(C)** Memory-guided saccade task. **(D)** Inhibition subtests of psychometric tests (see Supplementary Material for test description) **(E)** DTI. **(F)** Natural viewing task. **(G)** SVM-RFE. **(H)** Attentional eye traces and Tiled CNN.

In the ProSac and AntiSac tasks (25, 27), each trial started with the illumination of a central fixation point (FP) for 800–1,200 ms. The FP then disappeared and, after a gap period of 200 ms, a peripheral target appeared randomly at 10° to the left or right of the central FP. Participants had 1,000 ms to initiate and complete a saccade to the correct location and were instructed to look toward the target (ProSac, Figure 1A) or away from the target (AntiSac, Figure 1B). No error feedback was given. A single block of 60 ProSac and 60 AntiSac trials were obtained from each participant. In the MGSac task, participants were again instructed to maintain fixation at the central FP, after which two peripheral targets appeared sequentially for 100 ms each in one of four quadrants around the periphery of the screen. Participants were required to maintain fixation on the central FP for an additional 0, 600, 1,200, or 1,800 ms (randomly allocated) before receiving the go signal (disappearance of the FP). After the FP disappeared, participants were required to make two saccades as accurately as possible to the remembered locations of the peripheral targets in the same sequence in which they were presented (Figure 1C). A single block of 72 trials was collected for each participant (28).

The recordings were analyzed based on a set of measurements, and age corrections were applied (see Supplementary Material). For the ProSac task, 18 measurements were obtained, including percent correct trials, saccadic reaction time, saccade velocity (see Supplementary Material). The number of measurements for AntiSac and MGSac were 15 and 26, respectively. These measurements were treated as features for further classification analysis.

### Natural Viewing

Participants watched a series of five 1 min video clips, with each clip consisting of a sequence of uncorrelated short video snippets of 2–4 s duration chosen from a set of 70 snippets. Eye movements were recorded as described above of the right eye, and the participants were instructed to simply “watch and enjoy the clips” (Figure 1F).

To quantitatively gauge the visual properties of the scene elements looked at by each participant, the Itti-Koch saliency model (34, 35) was applied to each frame of the video clips to obtain the saliency maps of seven individual visual processing channels: color (C), intensity contrast (I), orientation (O), flicker (F), motion (M), line junction (J), and intensity variance (Var); two combination channels: CIOFM and CIOFMJ. Another top-down saliency map was derived from the Gaussian smoothed spatiotemporal gaze map of a group of healthy young adults (*n* = 19) from USC (30). The saliency model and its constituent visual channels have been previously validated, accurately predicting gaze patterns of controls watching natural videos, television, or playing video games (36, 37). Here, we used the same approach to objectively and quantitatively detect any differences in the saliency and visual properties of the scene elements looked at by children/youth with FASD vs. controls. The standardized values of the saliency maps were extracted at each recording point of the eye traces, resulting in what we call “attentional eye traces” throughout this paper. The 10-dimensional (seven visual channels, two combined channels, and one top-down) attentional eye traces obtained from each of the 70 snippets natural viewing assessment were treated as the data for the classification analysis (Figure 1H).

### Diffusion Tensor Imaging (DTI)

Brain imaging data were collected at 3 sites (Edmonton, AB, 1.5T Siemens Sonata; Kingston, ON, and Winnipeg, MB, both 3T Siemens Trio). All of the diffusion tensor images were acquired using a dual spin-echo echo planar imaging sequence. Manual deterministic tractography of the corpus callosum was performed in ExploreDTI (38) by a single operator, blinded to participant group, age, sex, and handedness. The corpus callosum was divided into 6 regions of interest (ROI): genu, rostral body, anterior midbody, posterior midbody, isthmus, and splenium from front to back (18, 39) (Figure 1E). Three eigenvalues, average length and angle were obtained for each ROI, and FA, MD, and perpendicular diffusivity (λ⊥) were calculated based on the eigenvalues averaged across all voxels in a given tract (see Supplementary Material). In total, 48 features were acquired from this DTI assessment (eight features for each ROI, and six ROIs in total).

### Psychometric Tests

The Developmental Neuropsychological Assessment, Second Edition (NEPSY-II) (40) is a standardized psychometric test battery for children 3–16 years of age. Data from five subtests involving attention and executive functioning (Figure 1D), memory and learning, and visuospatial processing were used in this study. In addition, subtests from the Working Memory Test Battery for Children that measure the verbal and visuospatial working memory, subtests from the Woodcock Johnson III test battery that measure applied problem solving and quantitative concepts, and a subtest from the Woodcock Reading Mastery Test that measures language and reading skills, were also used (see details in Supplementary Material). All subtests were standardized to have a mean standard score of 10 with a standard deviation of 3 (20). A total of 20 scores (features) were obtained from these tests for each participant.

### Single Assessment Data Analysis

The data from saccadic eye movements, DTI, and psychometric tests were analyzed using support vector machine-recursive feature elimination (SVM-RFE) (41). SVM-RFE allows group classification and feature selections at the same time. Data from participants who finished all six assessments were left out for testing and the remaining data was used for training with leave-one-out (LOO) as the cross validation method. The number of participants for training is summarized in Table 3. For the DTI assessment, since only a small number of training samples remained, and the FA, MD and perpendicular diffusivity were highly correlated with eigenvalues from which they were derived (see Supplementary Material), using all the features did not result in a good classifier. Therefore, a subsets of features (three eigenvalues, average length and angle of each of the six ROIs) were used as input.

**Table 3:** Participants for training single assessment classifiers.

	**ProSac**	**MGSac**	**AntiSac**	**Natural viewing**	**DTI**	**Psychometric**
n(FASD)	49	39	45	25	19	36
n(Control)	91	69	82	29	11	47

During training, data was first normalized to the 0–1 range within each feature dimension to avoid incorrect weighting among features. The normalized data was then analyzed with the SVM-RFE. For each iteration, the classification accuracy, and the contribution weight of each feature were computed, the least useful feature with the lowest contribution weight would be eliminated, and the remaining features became the input for the next iteration (Figure 1G). Classification accuracy, a set of useful features, and the corresponding classifier were the outputs for each iteration. The procedure terminated when no further feature was eliminated. The set of features and the classifier corresponding to the best classification accuracy were selected.

During testing, data was first normalized according to the range of the training data within each feature dimension. The trained classifier was then applied to the selected features of the test data set. Classification accuracy and the probability value of a participant being identified as having FASD were reported on this separate test set.

For the attentional eye trace data from the natural viewing experiment, two stacked tiled convolutional neural networks (Tiled CNNs, Figure 1H) (42) were used to learn a sparse representation of the data within each saliency channel. Each of the CNN was a topographic independent component analysis (TICA) network (43). The Tiled CNNs were pre-trained on a separate dataset obtained from young adults at USC. The learned representations for different saliency channels of the same video snippet were concatenated together (30).

A classification procedure was then applied to the learned representation of natural viewing data. During training, the input data was normalized to the 0–1 range across participants. A two-tail *t*-test with correction for multiple comparisons was used to reduce the input dimension by eliminating feature dimensions with no significant difference between FASD and controls. 70 weak classifiers were trained on data from each video snippet via L1-regularized logistic regression (LR), which is to solve the following problem (44):

$$\min\sum \left|w_{j}\right|+C\sum \text{log}(1+\exp\left(-y_{i}w^{T}x_{i}\right))$$

where *y*_*i*_ is the group label (0 for control and 1 for FASD), *x*_*i*_ is the input data of each participant, *w*_*j*_ is the regression weight of each input dimension and *C* is the parameter selected during cross validation. The 70 probabilities of being FASD after those classifiers were concatenated as a single vector input for training another LR classifier. LOO was used as the cross-validation method for this two-layer LR classifier training framework.

The test data for natural viewing were normalized across participants and filtered according to the training data before the classification. The classification accuracy and probability of a participant being identified as having FASD were then reported through the two-layer LR procedure.

### Multiple Assessments Data Classification

To take advantage of data from different assessments, we then performed a classification analysis on the dataset of the participants who finished all six assessments (*N* = 46, see Table 2). An iterative train-test procedure was applied. During each iteration: one participant's data set was excluded as test data; the remaining data were used for training with cross validation. The probability of a participant being identified as having FASD predicted from each assessment was concatenated as input for training a LR classifier. The iteration terminated when every participant's data was used as the test sample once and the accuracy was calculated based on the prediction labels acquired during testing.

### Multilinear Regression

Multilinear regression can be used to analyze the relationship of features and outcomes (differentiation probability of being FASD) across different assessments. The linear relationship between a response Y, which can be either the feature or the outcome of one assessment, and the regressors (explanatory variables) X, which are features from another assessment, for each observation, is measured by the following equation

$$E\left(Y|X\right)=\beta_{0}+\sum_{i\;=\;1}^{N}\beta_{i}x_{i}$$

where β_*i*_ is the coefficient. An estimation of Y, $\hat{\text{Y}}$, is obtained via X and $\hat{\beta }$, which was learned by minimizing the sum of squared residuals. The relationship is depicted by the square of the correlation coefficient r^2^ of Y and $\hat{\text{Y}}$. Features for saccadic eye movement and psychometric tests were the same as previously used in classification analysis. Differentiation probabilities of weak classifiers with non-zero regression weights (*N* = 35) were treated as features for natural viewing, and all features except λ⊥ were used for DTI. *F*-test was applied to decide the significance of regression.

### Performance Analysis of Classifiers

To statistically compare the performance of different classifiers, we first computed the performance variances by repeating the training and testing process 20 times for each classification method with a resampled training set. For every repetition, we performed a stratified resampling with replacement of the training set (maintaining the same number of samples in each group), trained the classifier, and tested on the test set. Twenty classification accuracies were acquired for each classifier. The average of the accuracies and their standard errors are shown in Figure S1. One-way ANOVA test was used to compare these accuracies of different classifiers, and the Tukey's honestly significant difference procedure was applied when multiple comparisons were involved. The significance level was set to be 0.05.

## Results

### Classification on Single Assessment

**Figure 2:**
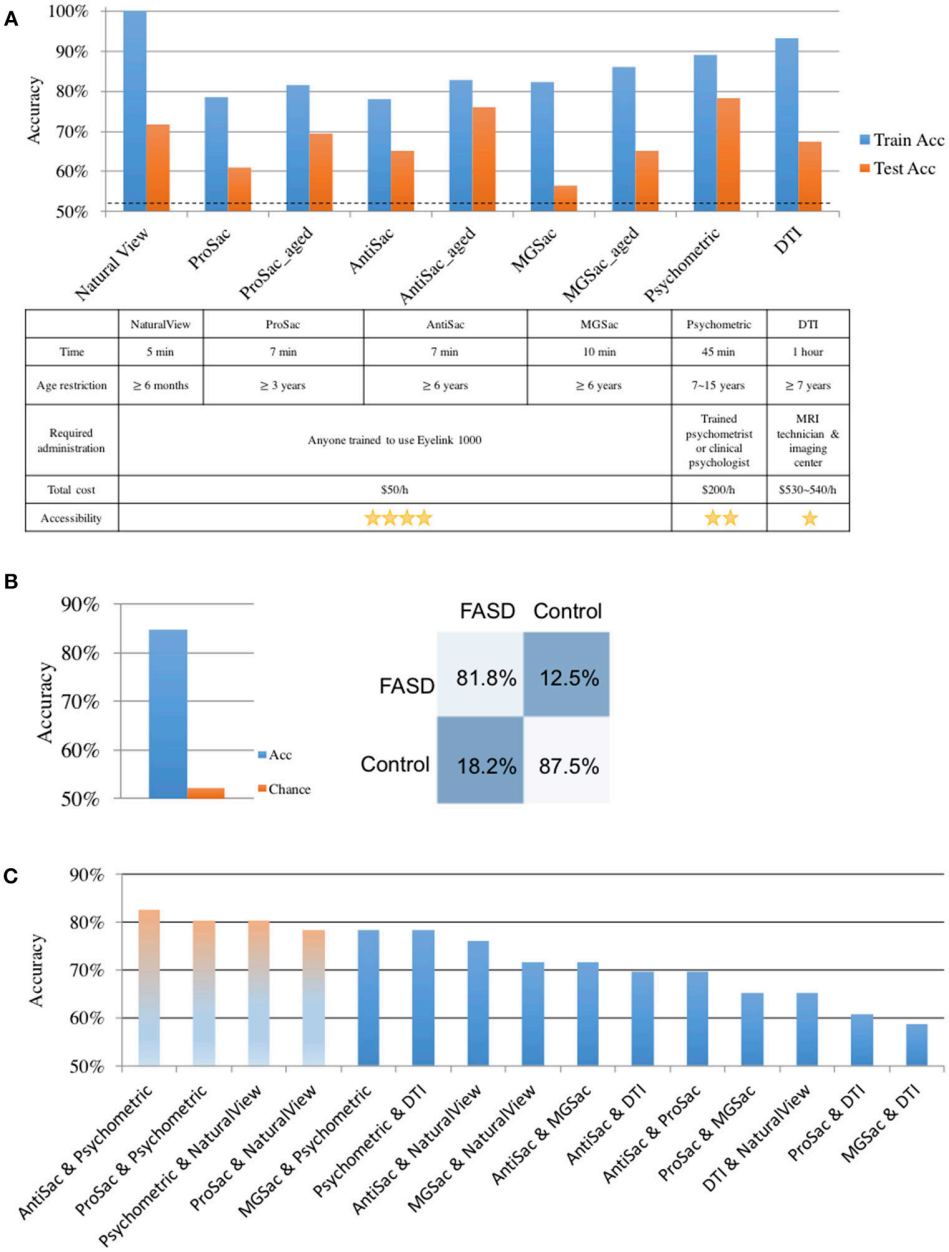
Classification results. **(A)** Classification accuracies and evaluations on single assessments. Top: train (cross validation) and test accuracies of each assessment. Bottom: evaluations of different assessments. **(B)** Classification accuracy on all assessments. Left: classification accuracy and the chance level. Right: the confusion matrix. **(C)** Classification results of pairwise assessments. The first three light-colored bars show accuracies of the top pairs. The forth shows the highest accuracy without psychometric tests.

First, we aimed to establish the classification accuracy obtained separately from each of the six assessments. Both cross validation accuracy on the training dataset and the classification accuracy on the test dataset are reported (Figure 2A). Chance level was 52.17%. To better understand the results of the feature elimination process, we report in Table 4 the features that were selected in the top-scoring classifier for each assessment taken individually. Their normalized contribution weights and categories are also summarized in Table 4. The contribution weights for the assessment features were normalized so that their absolute values summed to 1 (this normalization would not affect the relative ratio among features).

**Table 4:** Selected features and normalized weights for single assessment classification.

**Assessment**	**Feature**	**Weight**	**Category**
ProSac (age-corrected)	Percent of trials with step saccades	0.231	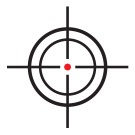
	Standard deviation of SRT for correct trials	0.176	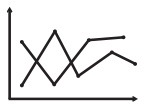
	Coefficient of variation of correct trials	0.145	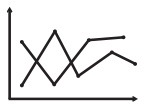
	Skew 2	0.086	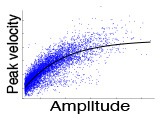
	Saccadic reaction time of correct trials	0.073	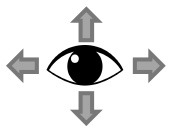
	Angle between direct path to target and first saccade	0.071	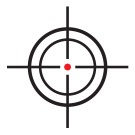
	Amplitude	0.070	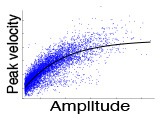
	Skew1	0.055	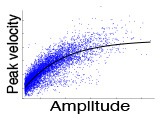
	Percent of direction errors that were corrected	0.043	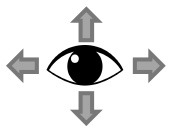
	Skew index	0.035	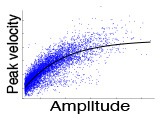
	Percent of express saccades(90-140ms)	0.015	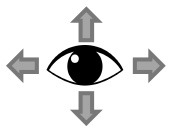
AntiSac (age-corrected)	Angle between direct path to target and first saccade	0.216	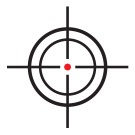
	Percent of anticipatory errors	0.174	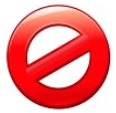
	Percent of correct trials	0.173	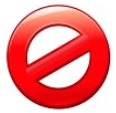
	Velocity	0.163	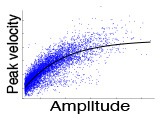
	Percent of direction errors	0.105	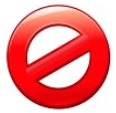
	Percent express saccades in both directions	0.060	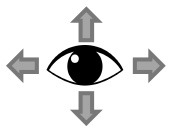
	Coefficient of variation of correct trials	0.056	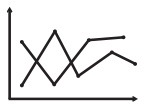
	Percent of direction errors that were corrected	0.042	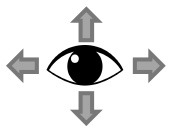
	Percent of trials with step saccades	0.010	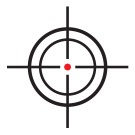
MGSac (age-corrected)	Percent of trials where they skipped the first target and go to second only	0.114	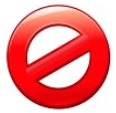
	Accuracy of the first saccade	0.110	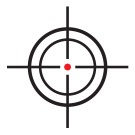
	Accuracy of the final fixation to the second target	0.089	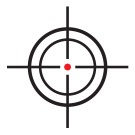
	Percent of timing errors	0.073	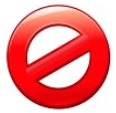
	Percent of All Timing Errors	0.070	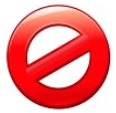
	Percent of false starts only	0.066	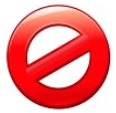
	Percent of trials that are sequence and timing errors	0.065	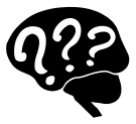
	Coefficient of variation of saccadic RT of first saccade of correct trials	0.064	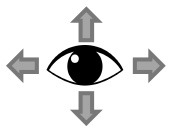
	Angle between direct path to target and first saccade	0.064	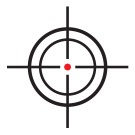
	Coefficient of variation of saccadic RT of second saccade of correct trials	0.063	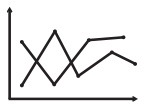
	Percent of sequence errors	0.063	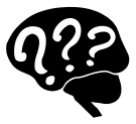
	Path length accuracy (actual/optimal path length)	0.053	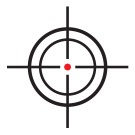
	Amplitude of the 2nd saccade	0.045	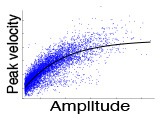
	Standard deviation of SRT of the 1st saccade of correct trials	0.037	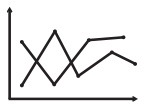
	SRT of the 1st saccade of correct trials	0.025	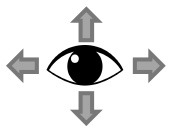
DTI	Genu: Length average	0.366	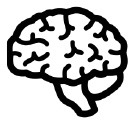
	Rostral: eigenvalue 3	0.290	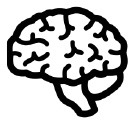
	Splenium: eigenvalue 3	0.150	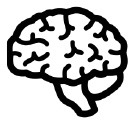
	Posterior: parallel diffusivity	0.081	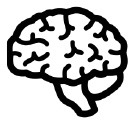
	Splenium: Length average	0.069	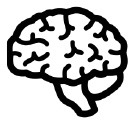
	Posterior: Angle average	0.044	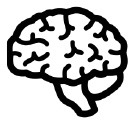
Psychometric	Quantitative concept standard score	0.408	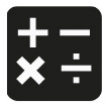 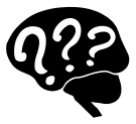
	Inhibition total errors scaled score	0.333	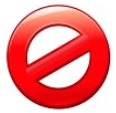
	Digit recall standard score	0.259	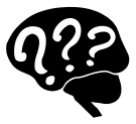

*
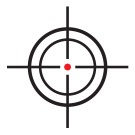
, Accuracy; 
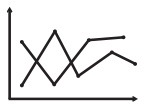
, Variability; 
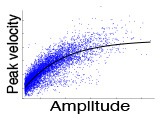
, Main sequence; 
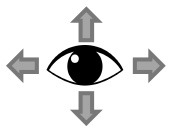
, other sensorimotor measures; 
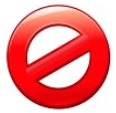
, Response inhibition; 
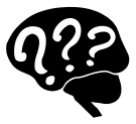
, Working memory; 
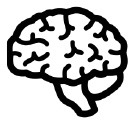
, Structure; 
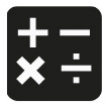
, Math; SRT, saccadic reaction time*.

For saccadic eye movement data, raw feature sets and age-corrected feature sets were used separately. Statistically significantly better accuracies were obtained using the age-corrected feature sets, indicating that the measurements varied with age. The numbers of selected features for age-corrected ProSac, AntiSac and MGSac were 11 out of 18, 9 out of 15, and 15 out of 26, yielding accuracies of 69.57, 76.09, and 65.22%, respectively. For clarity, the saccadic eye movement features were grouped into 6 main categories. Among these categories, features measuring accuracy, variability, main sequence (saccade amplitude, velocity, and duration), and other sensorimotor measures (including saccadic reaction time and express saccades) were common across all three types of tasks. AntiSac and MGSac have an additional feature category regarding response inhibition (direction errors and timing errors, respectively), and MGSac has an extra feature category for working memory (sequence errors). Features from all those categories contributed to differentiating the participants, with the most important (heaviest weighting) features coming from accuracy measurement (ProSac and AntiSac) and response inhibition measurement (MGSac).

A test accuracy of 71.74% was achieved for natural viewing. Among all the 70 video snippets, only 35 were selected by the LR classifier with statistically significant non-zero coefficients, indicating that eye movement recordings on half of the videos were not used and suggesting a possibly shorter natural viewing assessment (see Discussion).

A combination of measurements from the genu, rostral body, posterior midbody, and splenium of the corpus callosum contributed to the classifier of DTI, yielding a test accuracy of 67.39%.

The used features of the classifier for the psychometric data were scores of quantitative concepts (measuring math ability), inhibition, and digit recall (measuring working memory). The test accuracy reached 78.26%, which was the best single assessment classification accuracy. This finding was confirmed statistically using our classifier performance analysis procedure (see Supplementary section S5 and Figure S1A). However, eye movement features also resulted in good classification performance.

### Classification on Multiple Assessments

We hypothesized that the best classification accuracy would be obtained by combining features from multimodal assessments. Although this would be an expensive and resource-intensive approach, the combined accuracy can be used as a ceiling value for comparison with smaller subsets of assessments.

The classification accuracy based on data from all assessments under the iterative train-test procedure reached 84.78 with 52.17% as the chance level (naïve Bayes), which was an 11.4% improvement of the best single assessment accuracy. The sensitivity and specificity of the classification were 81.8 and 87.5%, respectively (Figure 2B). A further exploration of the feature combinations revealed that after dropping the MGSac and DTI features, the same result was still maintained. Thus, the optimized combination of assessments should be ProSac, AntiSac, natural viewing and psychometric tests.

Given this high combined accuracy, we next asked whether it could be approached with just pairs of two assessments. A similar iterative train-test classification procedure was thus applied to all possible pairs of assessments. The range of classification accuracies attained with different assessment pairs is summarized in Figure 2C. A combination of AntiSac and psychometric assessments achieved the highest pair-wise classification accuracy at 82.61%. The second highest accuracy was achieved by the combination of psychometric features and natural viewing (or ProSac) with a value of 80.43%. Our classifier performance analysis procedure further suggested no significant difference among the 5 top-scoring pairs, which are the 5 pairs that include the psychometric assessment (see Supplementary section 5). The highest accuracy without psychometric assessment was 78.26%, reached by the combination of ProSac and natural viewing, which was an improvement of 12.5 and 9.1% to their individual accuracy, respectively. Other combinations gave either non-improved or decreased accuracy compared with either individual assessment within the combination, suggesting that the combinations were noisy to each other.

### Multilinear Regression

**Figure 3:**
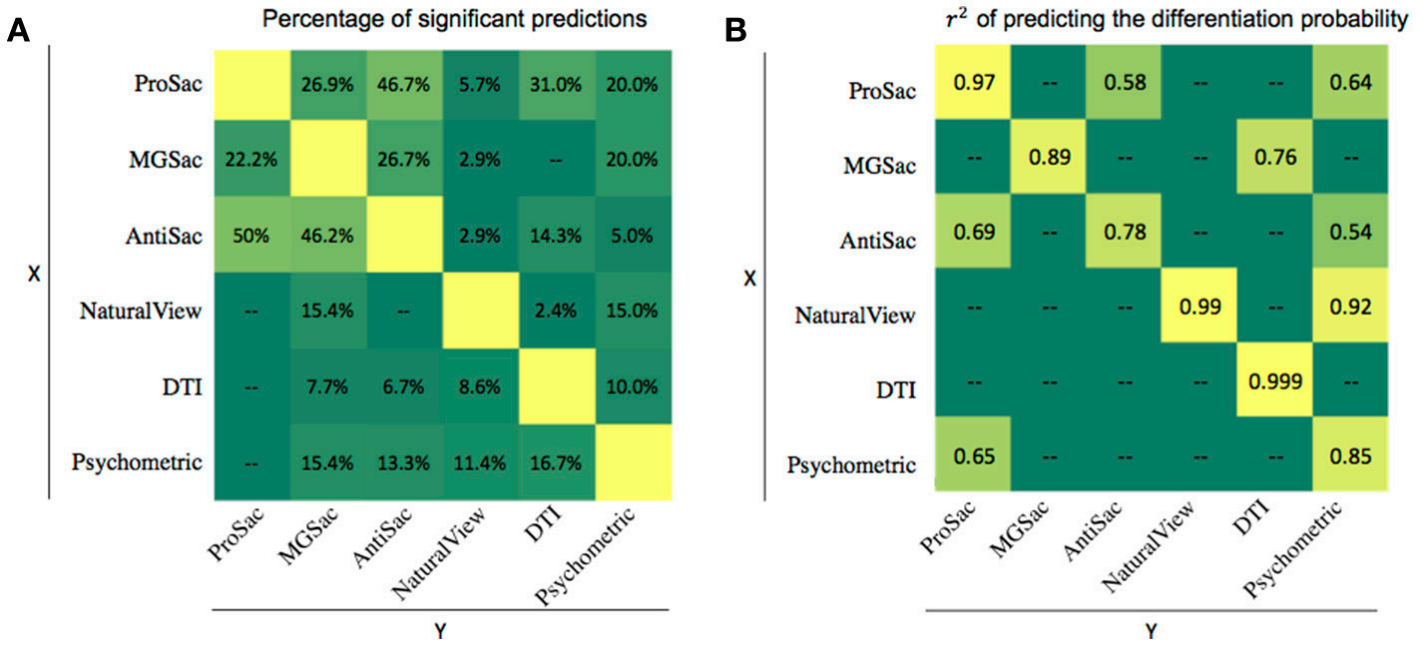
Multilinear regression results. **(A)** Ratio (in percentage) of significant features of one assessment predicted by another. **(B)** r^2^ of predicting the differentiation probability of one assessment from another.

If features collected in one assessment, or the outcome of that assessment, were predictable from features collected in another assessment, one of the two assessments could be eliminated, thus saving time and resources. To investigate this, we first counted the number of features within one assessment that could be significantly predicted (*p* < 0.05) by features from another, and then divided the number by the total number of features for that assessment (Figure 3A). All the percentages of significant predictions were below 50%. Therefore, all the assessments have their unique roles in analyzing the deficits of FASD, and the lack of full predictability is likely the reason why combined accuracy is best.

A second type of multilinear regression could be performed using measures from one assessment to predict the outcome from another. The r^2^ for significant predictions are summarized in Figure 3B. In most cases, the predictions were not significant. However, the Psychometric outcome could be predicted by natural viewing measures with an r^2^ of 0.92. This is also an explanation for the pairwise classification accuracy in that the ProSac-NaturalView pair achieved similar accuracy as the ProSac-Psychometric pair, indicating that the Psychometric and natural viewing assessments are interchangeable with respect to classification accuracy.

## Discussion

### General Findings

In this study, we showed that children with FASD could be distinguished from typically developing controls based on the measurements from a group of multidisciplinary tasks involving saccadic eye movements, natural viewing, psychometric tests, and DTI of the corpus callosum, while assessing their deficits during the classification procedure.

In the saccadic eye movement tasks, features derived from the accuracy of the saccade trajectory, the frequency of corrective saccades, the variability and the main sequence were always selected by the classifiers across the three tasks. Measurements of direction errors and percent of express saccades were significant in ProSac and AntiSac during classification. The saccadic reaction times were distinguishing in ProSac and MGSac. Inhibitory control was impaired for children with FASD in both AntiSac and MGSac, while measurements related to working memory in MGSac were also impacted. All of these findings were consistent with reported impairments in saccadic eye movement control in other studies (20, 21, 28, 45). However, instead of limiting the analysis to individual or a few eye movement parameters, the classifier uses all of the parameters as the input, and detects the distinguishing ones to predict the clinical group (FASD vs. control). Thus, a more comprehensive profile of parameters with differentiating significance (feature weights of the classifier) can be obtained, without any specific hypotheses about which features best distinguish the groups.

Our previous study showed that children with FASD could be classified through their natural viewing behaviors (30). Here we used LR instead of Adaboost, tested on a completely separate testing dataset about the same size of the training set, and achieved comparable classification accuracy. The LR also provided us with information about the significance among different video snippets, showing that half of them could be discarded with zero contribution to classifying this particular disorder. Upon casual visual inspection, and in terms of distribution of scene contents, filming location, and themes, the selected and non-selected video snippets showed no difference. We then measured the saliency model's response to each snippet as a possible differentiator, focusing on the peak saliency value in each frame as a measure of the most salient event or object in that frame. When ranking each video snippet by the average of the peak values across frames of every saliency channel (not including the top-down channel), and considering the top seven snippets (top 10% of all the snippets), the selected snippets by the classifier covered 6/7 for the flicker channel, 5/7 for the junction channel, and 5/7 for the two combined channels (CIOFM and CIOFMJ). This suggests that snippets with more dynamic and complex contents were more useful for classification. In addition, we computed the entropy of the top-down channel for each frame as a measure of inter-observer variability. All the snippets were ranked by the average entropy across frames. The snippets selected by the classifier covered 60% in the top 28 snippets (top 40% of all snippets). That is, snippets that elicited more varied viewing behavior, for example because they contained multiple actors as opposed to one, contributed more to classification. This result suggests an even more efficient experimental procedure of 2.5 min of natural viewing, or a re-consideration of our video snippet choices for a better classification result.

Performance scores relating to math ability, inhibition and working memory were selected by our elimination procedure to classify children with FASD from the typically developing children, with math ability measurement found to be the most distinguishing feature. Significantly poorer performances in these cognitive or executive functions have frequently been reported in the literature (20, 21, 45–49). For example, individuals with FASD were reported as having more difficulties in mathematics compared to other cognitive areas, and the deficit was correlated with the amount of prenatal alcohol exposure (50). These impairments were also correlated with abnormalities in various neural projections from the parietal and frontal lobes passing through the corpus callosum (50, 51).

DTI studies of the corpus callosum have consistently shown lower FA of the splenium of the FASD group (52–54), which is involved in visuospatial processing. Posterior regions of the corpus callosum including the posterior midbody, the isthmus, and the splenium are more affected. Altered FA or MD in the genu and isthmus have also been reported in the literature (55). Our classification results revealed abnormalities in the genu, the posterior midbody, and the splenium, with the posterior regions having two more contributing features. Although we also found significantly higher MD of splenium [one-way ANOVA, *F*_(1, 75)_ = 5.98, *p* = 0.017] for our participants with FASD, classifiers with FA or MD did not reach the best result. Instead, parallel diffusivity, the average length and the angle of the fibers were discovered to most contribute to classification accuracy, indicating more biometric features contribute to DTI abnormalities in the FASD group. We also found that involving the measurement from the rostral body, an anterior region that was not reported to be affected previously, increased classification performance significantly. Since a large portion of orbitofrontal fibers project through the rostral body (56), and visual processing was altered within the FASD group, our result may suggest a potential role of this anterior part of the corpus callosum in FASD that is not predicted simply by its FA or MD values.

### Classification Evaluation and Limitations

The best single assessment classification accuracy was achieved by the psychometric data. However, this may in part be due to the fact that these psychometric tests assess domains of function that are also part of the clinical diagnosis of our participants, including memory, executive functioning, abstract reasoning, and attention (9). Although the top feature selected by the classifier was the measurement of math ability, which was not directly included in the diagnostic procedure that provided ground truth for our sample, the scores for inhibition and digit recall (an indicator of working memory), might be a concern for confounding.

Another more important concern during classification is the generalizability of our methods. To address this, we used completely separated testing set/samples, and reported test accuracies on these independent testing set/samples. Cross-validation was applied during the training process, which is generally accepted as a good way to prevent overfitting and improve the generalizability. The classification accuracy is expected to improve if we have a larger dataset with more individuals assessed in the future, as the cross-validation accuracies reported on the training datasets were usually higher than the test accuracies. Besides the generalization problem of supervised learning, the high accuracy was achieved due to the relatively large training set (which included all but only one test sample at each time under the LOO condition).

The number of participants who finished all the six assessments is 46 (24 controls vs. 22 FASD), which is still larger than the dataset used for other studies about classification of children with FASD (13, 29). We used an iterative train-test procedure to make the maximum use of our data while considering its generalizability. A relatively simple classifier, the LR classifier, was used for our multiple assessments data. This classifier is easier to train, requiring much less computing time and resources, and can generalize better compared to other complicated classifier. More confidence could be added if we could have a larger number of participants, and recruit participants from other geographically different areas with more diverse background.

### Assessment Evaluation

The six assessments could be investigated from various aspects besides classification accuracy (Figure 2A). The natural viewing task only takes 5 min to run. The running time for saccadic eye movements are 7 min for ProSac and AntiSac, and 10 min for MGSac. The time required for psychometric tests and DTI are much longer, up to 1 h. Age restrictions apply according to either the participant's behavioral ability or the assessment requirements. Infants older than 2 months can do a natural viewing task (57). The age thresholds for saccadic eye movements are 3 years for ProSac and 6 years for AntiSac and MGSac. Children must be old enough to participate well in a brain imaging scan and the youngest participant who finished the scan was 7 years old. The subset of tests we used from the four psychometric batteries requires an overlapped age restriction between 5 to 15 years. With respect to the different paradigms, anyone who is trained to use the Eyelink 1000, which is just a laptop connected to a high-speed camera, can administrate the natural viewing and saccadic eye movement tasks. In contrast, a trained psychometrist or clinical psychologist is needed for the full battery of psychometric tests, while a brain imaging center and a MRI technician are required for DTI. The administration requirements make significant differences regarding the total assessment cost and accessibility. Natural viewing and saccadic eye movements are the lowest-cost assessments with the easiest accessibility. Brain imaging is the most expensive paradigm with the most difficult accessibility.

### Screening Procedure

**Figure 4:**
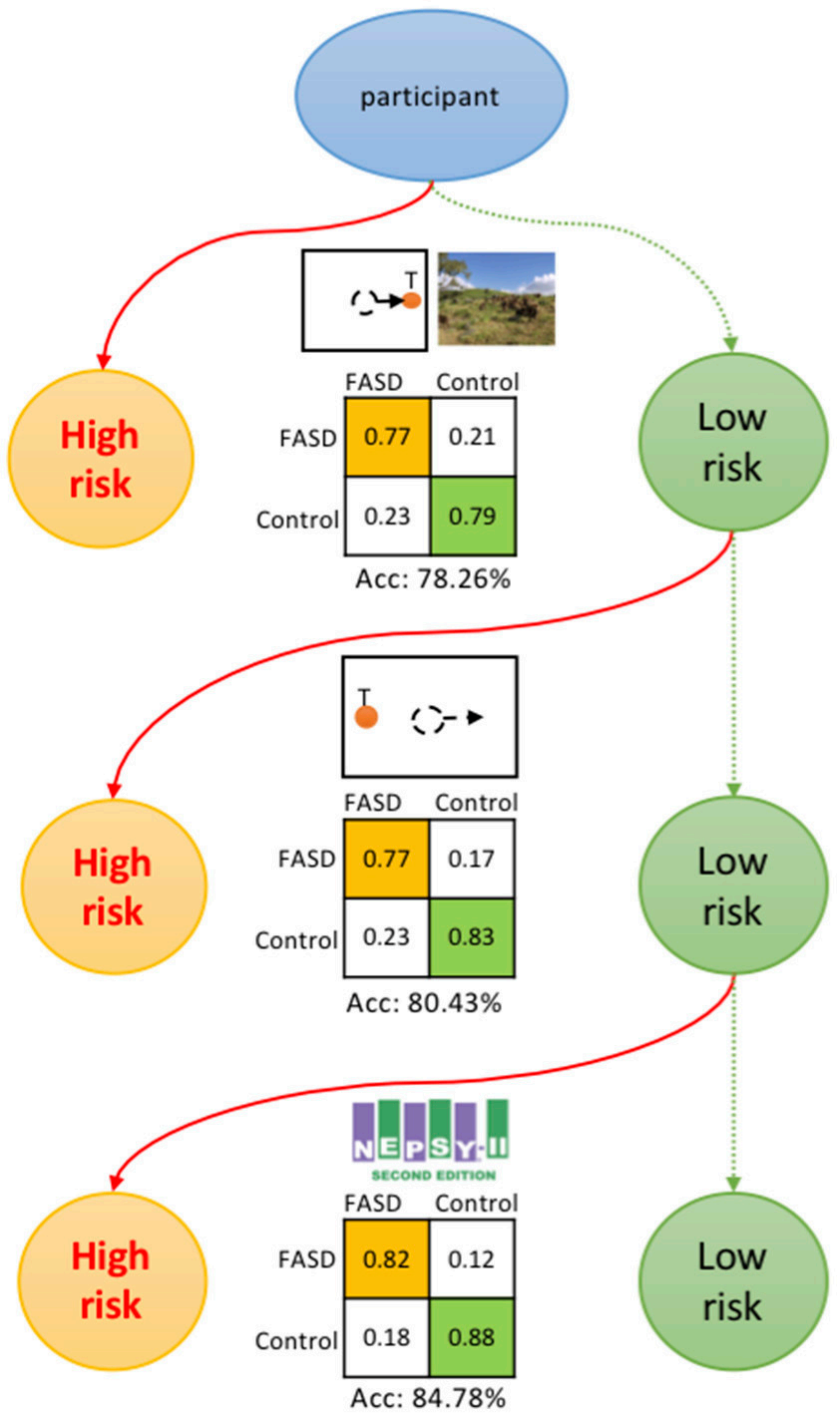
Screening procedure. The participant is first assessed by the ProSac and Natural viewing tasks, with an accuracy of 78.26% for estimating the risk of being FASD. For older children/youth, the addition of the AntiSac task and/or short battery of psychometric tests adds to the classification accuracy, adding greater confidence that at-risk children/youth are not missed. The confusion matrices are also shown.

A high-throughput and low-cost screening procedure could be proposed at this point (Figure 4). The participant is first assessed by the ProSac and Natural viewing tasks, with sensitivity 77.27%, specificity 79.17% and accuracy 78.26%, for estimating the risk of having FASD. If the participant is classified as high risk of having FASD (FASD score returned by the classifier higher than 0.55 on a 0 to 1 scale), then a full diagnostic evaluation could be suggested. This is the most cost-effective approach, with the least age restriction and task load, and can be further evaluated from a “value of information” perspective of view (32). For older children/youth, the addition of the AntiSac task and/or short battery of psychometric tests adds to the classification accuracy, adding greater confidence that at-risk children/youth are not missed.

**Figure 5:**
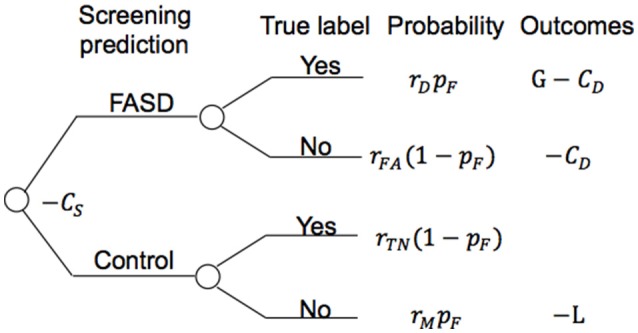
Model structure for cost evaluation of the screening procedure. *C*_*S*_, screening cost; *r*_*D*_, detection rate; *r*_*FA*_, false alarm rate; *r*_*TN*_, true negative rate; *r*_*M*_, miss rate; *p*_*F*_, *priori* probability of having FASD; *C*_*D*_, clinical diagnostic cost; G, gain; L, loss.

Figure 5 shows the model structure used for the annual cost-benefit analysis of the screening procedure for each individual. The probabilities in the model are derived from *a priori* probability of having FASD (*p*_*F*_, which could be the fraction of participants with FASD in the screened population), and the confusion matrix (detection rate *r*_*D*_, false alarm rate *r*_*FA*_, true negative rate *r*_*TN*_, and miss rate *r*_*M*_, see Figure 4 for those values of different screening procedures). A general cost of the screening is denoted as *C*_*S*_, of value smaller than $50 using initially only ProSac and Natural Viewing (Figure 2A). Adding the AntiSac task does not increase this value since it can be done together with ProSac and Natural Viewing. The cost of screening will increase by < $200 when a short battery of psychometric tests is also included. A participant predicted to be at high-risk of having FASD will have a further clinical diagnostic evaluation, which costs < $4,000 (excluding medication, hospitalization and other non-diagnostic costs) in both the United States and Canada (4, 58–60), denoted as *C*_*D*_. If our prediction of FASD is correct, then we will have a gain G for the early detection. Conversely, a loss L occurs when we miss a patient. The values for G and L are difficult to estimate, and no quantitative studies about them were found. However, a significant difference in the individual annual cost was reported for different severities of FASD (4). Early detection could mean that the progress of the disorder is mitigated by providing a supportive, enriched environment for the child, and access to services for the family, and the benefit gained from it could be at least $20,000 per year (difference of average annual health care costs between severe and mild FASD patients). Thus, G ≥ $20,000. If the screening procedure can be applied annually, then the cost of missing a patient, L, should be less than G, under the assumption that not all missed mild children/youth with FASD will develop severe symptoms within the year before the next screening. The expected value of the model for an individual is computed as follows:

$$\begin{array}{l} \text{EV}=r_{D}p_{F}\left(\text{G}-C_{D}-C_{S}\right)-r_{FA}\left(1-p_{F}\right)\left(C_{D}+C_{S}\right)\\ \;-r_{TN}\left(1-p_{F}\right)C_{S}-r_{M}p_{F}\left(\text{L}+C_{S}\right) \end{array}$$

For an individual with FASD, the expected annual savings with the aforementioned assumptions for the screening with eye movement tasks only is computed as:

$$\begin{array}{l} \text{EV}=0.77\left(\text{G}-C_{D}\right)-0.23\text{L}-C_{S}\\ \;\geq 0.77*(20000-4000)-0.23*20000-50=\$7670 \end{array}$$

The probability of missing a patient in a consecutive 4-year period is less than 0.3% (0.23^4^), which means that, with annual screening using a low-cost, high throughput procedure, a child/youth with FASD can be discovered with a probability higher than 99.7% within 4 years. Thus, the expected savings in 4 years is at least 7670(1 + 0.23 + 0.23^2^ + 0.23^3^) = $9933.

For our group of 46 children/youth with FASD in the testing group (with *p*_*F*_ = 0.48 in this group), the expected value of individual annual savings multiplied by 46 is $148,065 for a screening procedure composed of ProSac and Natural Viewing, $151892 with the addition of AntiSac, and $187220 with another addition of the three psychometric tests. Extrapolating this computation to a screened population of 1,000 individuals results in savings of more than 3 to 4 million dollars. If we have children with prenatal alcohol exposure or any suspected (e.g., by parents, caregivers or teachers) neurodevelopmental deficits to be screened, a reasonable estimation of *p*_*F*_ is likely to be higher than 0.48, leading to even more savings based on our model.

The proposed screening procedure consisting of three brief eye movement tasks and a substantially shortened set of 3 sub-tests from the psychometric battery achieved a mean sensitivity of ~82%, a mean specificity of ~88%, and an overall accuracy of ~85%, and can be administered in under 1.5 h. It provides a quantitative and objective signature of the disorder, for each individual, along multiple dimensions that encompass a range of cognitive and oculomotor functions, and alleviates the need for screening, or discrimination tools that rely on demographic, behavioral, physical, psychometric, face morphometric analysis, and history of maternal alcohol consumption (11, 14). Besides the benefits of time- and cost-effectiveness, the screening procedure is also much easier to implement with early or pre- school age children compared to the standard diagnostic process. The data can be analyzed through the same machine learning pipelines and thus all the screening estimations are achieved through the same standards. Such a screening procedure could be widely used at clinics, schools, or health units where young children are seen routinely, across different regions and areas, promoting communications within an interdisciplinary context. At-risk individuals, especially those with limited access to clinical resources, could receive a timely screening at a more affordable cost, and the disorder could potentially be detected at an earlier stage. Such an early screening that streamlines the referral and diagnostic process would have a substantial impact for children with FASD because it could lead to earlier intervention and treatment, which is crucial for neurodevelopmental disorders.

## Data Availability

The datasets for this study can be found here: https://web.gin.g-node.org/ccszhang/FASD_6_tests_data.

## Author Contributions

CZ: conception and design of work, analysis and interpretation of data, statistical analysis, drafting of the manuscript, revision of the manuscript. AP: acquisition of data, analysis of data, revision of the manuscript. P-HT: acquisition of data, analysis of data, revision of the manuscript. JR: conception and design of work, revision of the manuscript, obtaining funds, supervision. DM: conception and design of work, revision of the manuscript, supervision. LI: conception and design of work, revision of the manuscript, obtaining funds, supervision.

### Conflict of Interest Statement

The authors declare that the research was conducted in the absence of any commercial or financial relationships that could be construed as a potential conflict of interest.

## References

[1] Jones K Smith D. Recognition of the fetal alcohol syndrome in early infancy. Lancet (1973) 302:999–1001. 10.1016/S0140-6736(73)91092-1 4127281

[2] May PA Gossage JP Kalberg WO Robinson LK Buckley D Manning M. Prevalence and epidemiologic characteristics of FASD from various research methods with an emphasis on recent in-school studies. Dev Disabil Res Rev. (2009) 15:176–92. 10.1002/ddrr.68 19731384

[3] May PA Baete A Russo J Elliott AJ Blankenship J Kalberg WO. Prevalence and characteristics of fetal alcohol spectrum disorders. Pediatrics (2014) 134:855–66. 10.1542/peds.2013-3319 25349310 PMC4210790

[4] Stade B Ali A Bennett D Campbell D Johnston M Lens C. The burden of prenatal exposure to alcohol: revised measurement of cost. Can J Clin Pharmacol. (2009) 16:e91–102. 19168935

[5] Popova S Lange S Burd L Rehm J The Burden and Economic Impact of Fetal Alcohol Spectrum Disorder in Canada. Toronto, ON: Centre for Addiction and Mental Health (2015).

[6] Popova S Lange S Burd L Rehm J Burden and Social Cost of Fetal Alcohol Spectrum Disorders. Oxford Handbooks Online (2016).

[7] Benz J Rasmussen C Andrew G. Diagnosing fetal alcohol spectrum disorder: history, challenges and future directions. Paediatr Child Health (2009) 14:231–7. 10.1093/pch/14.4.231 20357921 PMC2690536

[8] Peadon E Elliott EJ. Distinguishing between attention-deficit hyperactivity and fetal alcohol spectrum disorders in children: clinical guidelines. Neuropsychiatr Dis Treat. (2010) 6:509–15. 10.2147/NDT.S7256 20856914 PMC2938300

[9] Chudley AE Conry J Cook JL Loock C Rosales T LeBlanc N. Fetal alcohol spectrum disorder: Canadian guidelines for diagnosis. Can Med Assoc J. (2005) 172:S1–S21. 10.1503/cmaj.1040302 15738468 PMC557121

[10] Goh YI Chudley AE Clarren SK Koren G Orrbine E Rosales T. Development of Canadian screening tools for fetal alcohol spectrum disorder. Can J Clin Pharmacol. (2007) 15:e344–66. 18840921

[11] Goh PK Doyle LR Glass L Jones KL Riley EP Coles CD. A decision tree to identify children affected by prenatal alcohol exposure. J Pediatr. (2016) 177:121–7. 10.1016/j.jpeds.2016.06.047 27476634 PMC5291174

[12] Burd L Klug MG Li Q Kerbeshian J Martsolf JT. Diagnosis of fetal alcohol spectrum disorders: a validity study of the fetal alcohol syndrome checklist. Alcohol (2010) 44:605–14. 10.1016/j.alcohol.2009.08.010 20053521

[13] Mutsvangwa TE Meintjes EM Viljoen DL Douglas TS Morphometric analysis and classification of the facial phenotype associated with fetal alcohol syndrome in 5-and 12-year-old children. Am J Med Genet A (2010) 152:32–41. 10.1002/ajmg.a.33137 20014122

[14] Kalberg WO May PA Blankenship J Buckley D Gossage JP Adnams CM. A practical testing battery to measure neurobehavioral ability among children with FASD. Int J Alcohol Drug Res. (2013) 2:51. 10.7895/ijadr.v2i3.83 25258654 PMC4170949

[15] Nuñez SC Roussotte F Sowell ER. Focus on: structural and functional brain abnormalities in fetal alcohol spectrum disorders. Alcohol Res Health (2011) 34:121–31. 23580049 PMC3860550

[16] Fryer SL Schweinsburg BC Bjorkquist OA Frank LR Mattson SN Spadoni AD. Characterization of white matter microstructure in fetal alcohol spectrum disorders. Alcohol Clin Exp Res. (2009) 33:514–21. 10.1111/j.1530-0277.2008.00864.x 19120066 PMC2651988

[17] Green CR Lebel C Rasmussen C Beaulieu C Reynolds JN. Diffusion tensor imaging correlates of saccadic reaction time in children with fetal alcohol spectrum disorder. Alcohol Clin Exp Res. (2013) 37:1499–507. 10.1111/acer.12132 23551175

[18] Paolozza A Treit S Beaulieu C Reynolds JN. Response inhibition deficits in children with fetal alcohol spectrum disorder: relationship between diffusion tensor imaging of the corpus callosum and eye movement control. Neuroimage Clin. (2014) 5:53–61. 10.1016/j.nicl.2014.05.019 24967159 PMC4066187

[19] Mattson SN Crocker N Nguyen TT. Fetal alcohol spectrum disorders: neuropsychological and behavioral features. Neuropsychol Rev. (2011) 21:81–101. 10.1007/s11065-011-9167-9 21503685 PMC3410672

[20] Paolozza A Rasmussen C Pei J Hanlon-Dearman A Nikkel SM Andrew G. Deficits in response inhibition correlate with oculomotor control in children with fetal alcohol spectrum disorder and prenatal alcohol exposure. Behav Brain Res. (2014) 259:97–105. 10.1016/j.bbr.2013.10.040 24185031

[21] Paolozza A Rasmussen C Pei J Hanlon-Dearman A Nikkel SM Andrew G. Working memory and visuospatial deficits correlate with oculomotor control in children with fetal alcohol spectrum disorder. Behav Brain Res. (2014) 263:70–9. 10.1016/j.bbr.2014.01.024 24486257

[22] Mattson SN Riley EP Delis DC Stern C Jones KL. Verbal learning and memory in children with fetal alcohol syndrome. Alcohol Clin Exp Res. (1996) 20:810–6. 10.1111/j.1530-0277.1996.tb05256.x 8865953

[23] Mattson SN Riley EP A review of the neurobehavioral deficits in children with fetal alcohol syndrome or prenatal exposure to alcohol. Alcohol Clin Exp Res. (1998) 22:279–94. 10.1111/j.1530-0277.1998.tb03651.x 9581631

[24] Kowler E. Eye movements: the past 25 years. Vision Res. (2011) 51:1457–83. 10.1016/j.visres.2010.12.014 21237189 PMC3094591

[25] Munoz DP Everling S Look away: the anti-saccade task and the voluntary control of eye move- ment. Nat Rev Neurosci. (2004) 5:218–28. 10.1038/nrn1345 14976521

[26] Baluch F Itti L. Mechanisms of top-down attention. Trends Neurosci. (2011) 34:210–24. 10.1016/j.tins.2011.02.003 21439656

[27] Coe BC Munoz DP. Mechanisms of saccade suppression revealed in the anti-saccade task. Philos Trans R Soc Lond B Biol Sci. (2017) 372:20160192. 10.1098/rstb.2016.0192 28242726 PMC5332851

[28] Paolozza A Titman R Brien D Munoz DP Reynolds JN. Altered accuracy of saccadic eye movements in children with fetal alcohol spectrum disorder. Alcohol Clin Exp Res. (2013) 37:1491–8. 10.1111/acer.12119 23578065

[29] Tseng PH Cameron IG Pari G Reynolds JN Munoz DP Itti L. High-throughput classification of clinical populations from natural viewing eye movements. J Neurol. (2013) 260:275–84. 10.1007/s00415-012-6631-2 22926163

[30] Tseng PH Paolozza A Munoz DP Reynolds JN Itti L Deep learning on natural viewing behaviors to differentiate children with fetal alcohol spectrum disorder. In: Yin H Tang K Gao Y Klawonn F Lee M Li M Weise T Yao W editors. Intelligent Data Engineering and Automated Learning-IDEAL 2013. Hefei: Springer (2013). p. 178–85.

[31] Wang S Jiang M Duchesne XM Laugeson EA Kennedy DP Adolphs R. Atypical visual saliency in autism spectrum disorder quantified through model-based eye tracking. Neuron (2015) 88:604–16. 10.1016/j.neuron.2015.09.042 26593094 PMC4662072

[32] Feltham GA The value of information. Account Rev. (1968) 43:684–96.

[33] Reynolds JN Weinberg J Clarren S Beaulieu C Rasmussen C Kobor M. Fetal alcohol spectrum disorders: gene-environment interactions, predictive biomarkers, and the relationship between structural alterations in the brain and functional outcomes. Semin Pediatr Neurol. (2011) 18:49–55. 10.1016/j.spen.2011.02.006 21575841 PMC4930322

[34] Itti L Koch C Niebur E A model of saliency-based visual attention for rapid scene analysis. IEEE Trans Pattern Anal Mach Intell. (1998) 11:1254–9. 10.1109/34.730558

[35] Itti L Koch C. A saliency-based search mechanism for overt and covert shifts of visual attention. Vision Res. (2000) 40:1489–1506. 10.1016/S0042-6989(99)00163-7 10788654

[36] Carmi R Itti L. Visual causes versus correlates of attentional selection in dynamic scenes. Vision Res. (2006) 46:4333–45. 10.1016/j.visres.2006.08.019 17052740

[37] Peters RJ Itti L Applying computational tools to predict gaze direction in interactive visual environments. ACM Trans Appl Percept. (2008) 5:9 10.1145/1279920.1279923

[38] Leemans A Jeurissen B Sijbers J Jones D ExploreDTI: a graphical toolbox for processing, analyzing, and visualizing diffusion MR data. In: 17th Annual Meeting of International Society for Magnetic Resonance in Medicine Honolulu, HI (2009). p. 3537.

[39] Witelson SF. Hand and sex differences in the isthmus and genu of the human corpus callosum: a postmortem morphological study. Brain (1989) 112:799–835. 10.1093/brain/112.3.799 2731030

[40] Korkman M Kirk U Kemp S NEPSY-II: A Developmental Neuropsychological Assessment. San Antonio, TX: Harcourt Assessment (2007).

[41] Guyon I Weston J Barnhill S Vapnik V Gene selection for cancer classification using support vector machines. Mach Learn. (2002) 46:389–422. 10.1023/A:1012487302797

[42] Ngiam J Chen Z Chia D Koh PW Le QV Ng AY Tiled convolutional neural networks. In: Advances in Neural Information Processing Systems Vancouver (2010). p. 1279–87.

[43] Hyvärinen A Hoyer P Inki M. Topographic independent component analysis. Neural Comput. (2001) 13:1527–58. 10.1162/089976601750264992 11440596

[44] Fan RE Chang KW Hsieh CJ Wang XR Lin CJ LIBLINEAR: a library for large linear classification. J Mach Learn Res. (2008) 9:1871–4. 10.1145/1390681.1442794

[45] Rasmussen C. Executive functioning and working memory in fetal alcohol spectrum disorder. Alcohol Clin Exp Res. (2005) 29:1359–67. 10.1097/01.alc.0000175040.91007.d0 16131842

[46] Fryer SL Tapert SF Mattson SN Paulus MP Spadoni AD Riley EP Prenatal alcohol expo- sure affects frontal–striatal BOLD response during inhibitory control. Alcohol Clin Exp Res. (2007) 31:1415–24. 10.1111/j.1530-0277.2007.00443.x 17559542

[47] Lebel C Rasmussen C Wyper K Andrew G Beaulieu C. Brain microstructure is related to math ability in children with fetal alcohol spectrum disorder. Alcohol Clin Exp Res. (2010) 34:354–63. 10.1111/j.1530-0277.2009.01097.x 19930234

[48] Rasmussen C Bisanz J The relation between mathematics and working memory in young children with fetal alcohol spectrum disorders. J Spec Educ. (2011) 45:184–91. 10.1177/0022466909356110

[49] Rasmussen C Tamana S Baugh L Andrew G Tough S Zwaigenbaum L. Neuropsychological impairments on the NEPSY-II among children with FASD. Child Neuropsychol. (2013) 19:337–49. 10.1080/09297049.2012.658768 22384972

[50] Rasmussen C Bisanz J Exploring mathematics difficulties in children with fetal alcohol spectrum disorders. Child Dev Perspect. (2009) 3:125–30. 10.1111/j.1750-8606.2009.00091.x

[51] Wozniak JR Muetzel RL Mueller BA McGee CL Freerks MA Ward EE. Microstructural corpus callosum anomalies in children with prenatal alcohol exposure: an extension of previous diffusion tensor imaging findings. Alcohol Clin Exp Res. (2009) 33:1825–35. 10.1111/j.1530-0277.2009.01021.x 19645729 PMC2895908

[52] Ma X Coles CD Lynch ME LaConte SM Zurkiya O Wang D. Evaluation of corpus callosum anisotropy in young adults with fetal alcohol syndrome according to diffusion tensor imaging. Alcohol Clin Exp Res. (2005) 29:1214–22. 10.1097/01.ALC.0000171934.22755.6D 16046877

[53] Sowell ER Mattson S Thompson P Jernigan T Riley E Toga A. Mapping callosal morphology and cognitive correlates Effects of heavy prenatal alcohol exposure. Neurology (2001) 57:235–44. 10.1212/WNL.57.2.235 11468307

[54] Sowell ER Johnson A Kan E Lu LH Van Horn JD Toga AW. Mapping white matter integrity and neurobehavioral correlates in children with fetal alcohol spectrum disorders. J Neurosci. (2008) 28:1313–9. 10.1523/JNEUROSCI.5067-07.2008 18256251 PMC3567846

[55] Wozniak JR Mueller BA Chang PN Muetzel RL Caros L Lim KO. Diffusion tensor imaging in children with fetal alcohol spectrum disorders. Alcohol Clin Exp Res. (2006) 30:1799–806. 10.1111/j.1530-0277.2006.00213.x 17010147 PMC2895767

[56] Huang H Zhang J Jiang H Wakana S Poetscher L Miller MI. DTI tractography based parcellation of white matter: application to the mid-sagittal morphology of corpus callosum. Neuroimage (2005) 26:195–205. 10.1016/j.neuroimage.2005.01.019 15862219

[57] Jones W Klin A. Attention to eyes is present but in decline in 2–6-month-old infants later diagnosed with autism. Nature (2013) 504:427–31. 10.1038/nature12715 24196715 PMC4035120

[58] Lupton C Burd L Harwood R. Cost of fetal alcohol spectrum disorders. Am J Med Genet C Semin Med Genet. (2004) 127C:42–50. 10.1002/ajmg.c.30015 15095471

[59] Stade B Ungar WJ Stevens B Beyene J Koren G The burden of prenatal exposure to alcohol: measurement of cost. J FAS Int. (2006) 4:1–14.

[60] Popova S Lange S Burd L Chudley AE Clarren SK Rehm J. Cost of fetal alcohol spectrum disorder diagnosis in Canada. PLoS ONE (2013) 8:e60434. 10.1371/journal.pone.0060434 23593216 PMC3617033

