# Supplementary material for: Detection of Children/Youth With Fetal Alcohol Spectrum Disorder Through Eye Movement, Psychometric, and Neuroimaging Data
Source: Front Neurol. 2019 Feb 18;10:80. doi: 10.3389/fneur.2019.00080 (PMC6388649; doi:10.3389/fneur.2019.00080)
Supplement: Supplementary file 1 [file Data_Sheet_1.docx]

Supplementary Material

Detection of Children/Youth with Fetal Alcohol Spectrum Disorder through Eye Movement, Psychometric and Neuroimaging Data

Chen Zhang, Angelina Paolozza, Po-He Tseng, James N. Reynolds, Douglas P. Munoz, Laurent Itti*

*** Correspondence:** Laurent Itti: itti@usc.edu

# Diagnostic Criteria

Children with three neurological conditions are involved as the FASD group in this study: Fetal Alcohol Syndrome (FAS), partial FAS (pFAS) and Alcohol Related Neurodevelopmental Disorder (ARND). The diagnosis of FAS requires the presence of (i) facial abnormalities; (ii) impaired pre- and/or postnatal growth; and (iii) abnormal central nervous system (CNS) or neurobehavioral disorders. A diagnosis of FAS can be made in the absence of a confirmed history of prenatal alcohol exposure. The diagnostic criteria for pFAS include the presentation of the special craniofacial and physical features, impaired CNS and/or neurobehavioral disorders not explained by other causes, and a confirmed history of maternal alcohol consumption. The diagnosis of ARND is made when the child presents structural and/or functional abnormalities of the CNS excluding other diagnoses with confirmed maternal alcohol exposure, but few or no physical features (1).

# Saccadic eye movements

Saccadic eye movements are the rapid shifts that redirect the line of sight to bring new locations to the fovea (2), where the density of photoreceptors is greatest. Images that fall on the fovea are processed with high visual acuity. The distance and direction of a new target image from the current gaze location are processed by the saccadic system and a high-velocity eye movement is generated to bring the target to the fovea (3). Saccades are the natural scanning movements of the eyes that occur about three to four times per second (4), making the sampling of the visual environment and the allocation of the visual attention in efficient ways (5).

Saccadic eye movements include both the reflex (or spontaneous) and voluntary types (6). Reflex saccades occur when a novel stimulus appears or during the scanning of visual scenes. They can be recorded during the natural viewing task. Voluntary (or internally generated) saccades are made of purposes, including the prosaccades, which are saccades towards a peripheral target; the antisaccades, which are saccades directed to the opposite of a peripheral target; and the memory-guide (or remembered) saccades, which are saccades made to the remembered location(s) of the target(s).

During the prosaccade, antisaccade and memory-guided saccade tasks, trials where the participant didn’t fixate appropriately or eye tracking was lost were removed. The rest of the trials are used for data analysis as viable trials. A standardized t-score equation for age correction was obtained for each age on the control group, and the age-corrected scores were calculated based on the equation for the FASD group.

## Prosaccade task

The prosaccade task is the simplest eye movement task in this study only involving basic sensorimotor integration. In this task, the subject was first presented a fixation point (FP) in the center of the screen. The FP disappeared about 200ms before the peripheral target appeared which was 10 degrees to the left or the right of the FP. Measurements of the prosaccade behaviors include saccadic reaction time, performance accuracy, variability and parameters related to the main sequence (See Table S1 for details). Express saccades which are automatically generated with very short reaction time occur after about 90-120ms of the appearance of the target during the task. Inaccuracies can be recorded when the target is overshot or undershot. Corrective saccades are then made to adjust the eye to the desired position. Anticipatory saccades are those generated less than 90ms after the appearance of the target. The threshold of 90ms was chosen as the approximate neural processing latency of the saccadic system. Direction errors occur when the initial saccade was directed away from the target.

## Antisaccade task

The requirement of suppression of involuntary saccades in the antisaccade task makes the measurements of the task involving not only the sensorimotor functions but also the ability of inhibition. In this task, the subject was asked to first fixate on the FP, and then look to the opposite side when a peripheral targets appeared 10 degrees to the left or the right of the FP. Direction errors occurred when the subject failed to inhibit the automatic saccade towards the peripheral target. Direction errors occur when the initial saccade was directed towards the target.

## Memory-guided saccade task

The memory-guided task is another voluntary saccadic eye movement task which measures the sensorimotor function, inhibition ability and working memory. The subject in this task was asked to fixate on the FP while two peripheral targets flashed in sequence, and make two saccades towards the target locations according to their appearance order after the disappearance of the FP. The gap between the flashing of targets and the disappearance of the FP varied randomly for 0, 600, 1200, 1800ms. Sequence errors occurred when the subject didn’t follow the target order, and initiated the saccade towards the second target rather than the first. Timing errors occurred if saccades started less than 90ms after the disappearance of the FP.

# Diffusion tensor imaging (DTI)

The diffusion tensor imaging in this study was used to measure the white matter connectivities in the corpus callosum. Acquisition of the DTI data only requires the structural MRI, which thus makes the test task-free.

DTI measures the three-dimensional orthogonal diffusion directions of the water molecules within the white matter. The orientations of diffusion are depicted by three eigenvalues λ_1_, λ_2_ and λ_3_, with λ_1_ to be the largest and is recognized as the primary diffusion direction. Since λ_1_ represents the diffusivity parallel to axons, it’s also called parallel or axial diffusivity. The average of λ_2_ and λ_3_ is orthogonal to the axons, and is known as the perpendicular diffusivity. Other measurements for each of the corpus callosum regions are the average length, the average angle, the mean diffusivity (MD) and the fractional anisotropy (FA), which are defined as the following:

# Psychometric assessments

The NEPSY-II (7) is specially designed for children aged 3 to 16 years old regarding their neuropsychological, intellectual, memory and achievement performances and can assist the identification of cognitive deficits related to neurodevelopmental disorders. This battery assesses multiple domains of attention and executive functioning, memory and learning, sensorimotor functioning, social perception, language, and visuospatial processing. The NEPSY- II returns raw, standardized scores and percentile rankings based on age.

The animal sorting subtest was used to assess the ability of formulating basic concepts, transferring them into categories and shifting between concepts. Functioning working memory is also involved in this subtest. The child was instructed to sort cards into two groups of four based on his/her own sorting criteria. Errors occurred when the child made a wrong sort or repeated a sort. The age restriction of this subtest is from 7 to 16 years old.

The auditory attention (AA) and response set (RS) subtest contains two parts. The AA part was designed to test the selective auditory attention and vigilance. The child listened to a series of words and was instructed to touch the appropriate circle when a target word appeared. The RS part was used to assess the ability of attention shifting and maintaining. The child would touch a circle either match or contrast to the stimuli according to the instruction. This part involves the ability to inhibit the previously learned responses and respond appropriately to stimuli. Errors occurred when the child failed to make a response, made an incorrect response, or failed to make an inhibitory response as needed. The age restriction is 5 to 16 for AA and 7 to 16 for RS.

The inhibition subtest was chosen to test the ability of inhibition of automatic responses and switching between response types. The child was instructed to name either the shape or direction, or to make an opposite response, of a series of black and white shapes or arrows, or switch between correct or alternate response according to the colors. Errors occurred when the child provided an incorrect response. The age restriction is 5 to 16 years old.

The subtest of memory of names was selected to assess the ability of learning names, retaining them in short-term and long-term (delayed task) memory. The child needed to memorize the name of six or eight children drawn on cards while being read their names, and recall the name when shown the card again. The age restriction is from 5 to 16 years old for this subtest.

Arrows is the subtest to assess the visuospatial processing ability of line orientation. The child was shown an array of arrows surrounding a target and instructed to point out the arrow(s) pointing to the center of the target. The age restriction is 5 to 16 years old.

The scaled scores are typically the primary scores indicating the global aspects or key clinical variables of the subtests, or the contrast scores if multiple primary scores are offered by a subtest. The combined scores are the total scores of two measures within a subtest.

The Working Memory Test Battery for Children (WMTB-C) is designed for the assessment of working memory for children in 5 to 15 years. Two out of nine subtests were used in this study. The digit recall is designed to test verbal working memory as for the phonological loop aspect, involving holding verbal information for a short period of time; while the block recall is designed for spatial working memory as for the visuospatial sketchpad aspect, involving holding visual and spatial information (8). During the digit recall session, the child was read a string of numbers and instructed to repeat the numbers in their read order. During the block recall session, the child was shown a sequence of tapped blocks and instructed to repeat the sequence. In both of the subtests, the task ended when the child makes three errors in a row, and the number and span of correct trials were recorded.

The Woodcock Johnson III tests assess various cognitive abilities regarding mathematics, comprehension, language, memory and so on. The subtest chosen for this study is the Applied Problems and Quantitative Concepts. Applied Problems requires the construction of mental models to solve problems through the application of insight or quantitative reasoning. Solutions to these problems require access to complex cognitive processes and the calculation abilities that depend on them. Many Applied Problems items involve language comprehension (i.e., either listening ability or reading comprehension), and tasks are sometimes performed mentally using the visual working memory process. Quantitative reasoning is also required in addition to mathematical knowledge.

The Woodcock Reading Mastery Test-revised (WRMT) is selected to test the reading ability. The word identification test measures one of the basic reading skills: reading and pronouncing words in isolation. This test has no age restrictions for children.

Table S1. Measurements of Prosaccade

| Index | Measurement | Description | Category |
| --- | --- | --- | --- |
| 1 | % of correct trials | Number of correct trials divided by number of viable trials |  |
| 2 | SRT of correct trials | Interval between the target appearance and saccade onset for correct trials | 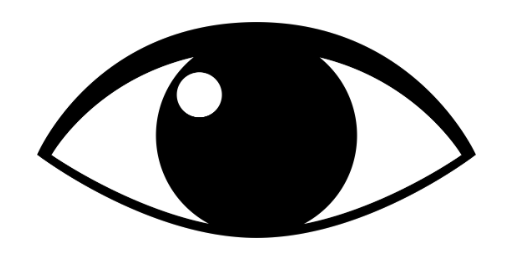 |
| 3 | SD of SRT for correct trials | Standard deviation of the SRT representing the amount of variation within an individual’s reaction time |  |
| 4 | Coefficient of variation of correct trials | Relative variability of the SRT within an individual’s reaction time |  |
| 5 | % of express saccades | Number of express saccades (90-120ms after target onset) divided by number of viable trials | 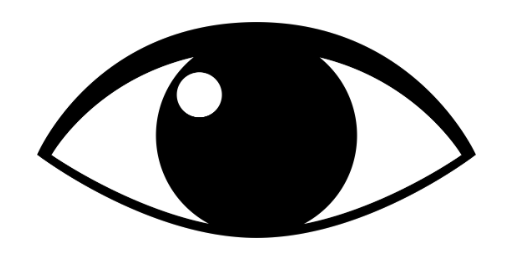 |
| 6 | % of express saccades in both directions | Number of express saccades in both directions divided by number of viable trials | 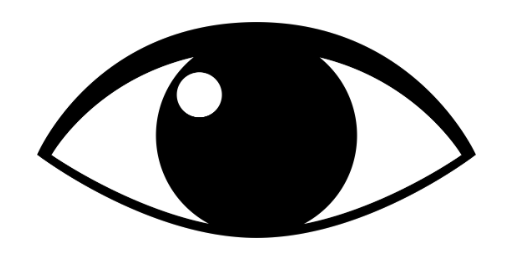 |
| 7 | % of direction errors | Number of direction errors divided by number of viable trials |  |
| 8 | % of anticipatory errors | Number of anticipatory errors divided by number of viable trials | 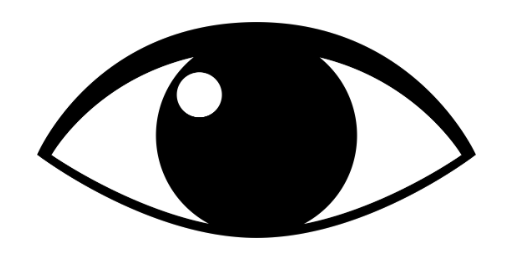 |
| 9 | % of trials with step saccades | Number of trials with step saccades divided by number of viable trials |  |
| 10 | Velocity | Speed of the saccade |  |
| 11 | Amplitude | The distance of the saccade |  |
| 12 | Angle between direct path and 1^st^ saccade | Angle between the line from the fixation point to the target and the line from the 1^st^ saccade starting point to endpoint |  |
| 13 | Duration | Interval between saccade onset and offset |  |
| 14 | Deceleration | Slope of velocity from peak to saccade termination |  |
| 15 | Acceleration | Slope of velocity from saccade onset to peak |  |
| 16 | Skew 1 | Slope of the mean acceleration phase of the velocity profile |  |
| 17 | Skew 2 | Slope of the mean deceleration phase of the velocity profile |  |
| 18 | Skew index | (slope1-slope2)/(slope1+slope2) |  |

%: percent; CT: correct trials; SRT: saccadic reaction time; SD: standard deviation; slope1: mean acceleration; slope2: mean deceleration

: Accuracy; : Variability; : Main sequence; : other sensorimotor measures


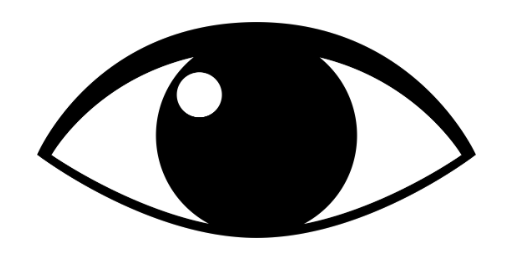


Table S2. Measurements of Antisaccade

| Index | Measurement | Description | Category |
| --- | --- | --- | --- |
| 1 | % of correct trials | Number of correct trials divided by number of viable trials | 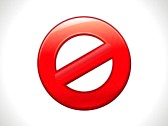 |
| 2 | SRT of correct trials | Interval between the target appearance and saccade onset for correct trials | 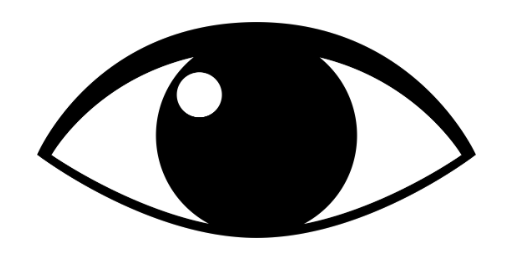 |
| 3 | SD of SRT for correct trials | Standard deviation of the SRT representing the amount of variation within an individual’s reaction time |  |
| 4 | Coefficient of variation of correct trials | Relative variability of the SRT within an individual’s reaction time |  |
| 5 | % of express saccades in both directions | Number of express saccades in both directions divided by number of viable trials | 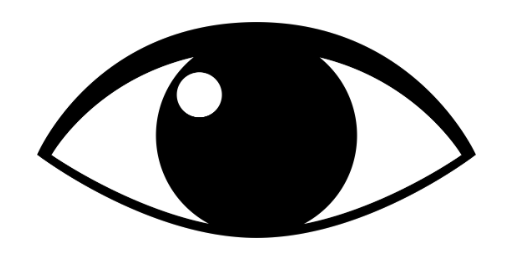 |
| 6 | % of express saccades | Number of express divided by number of viable trials | 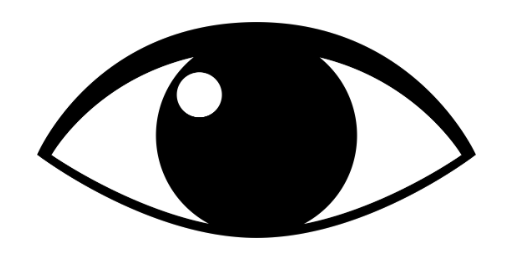 |
| 7 | SRT of direction errors | Interval between the target appearance and the onset of a wrong-directed initial saccade | 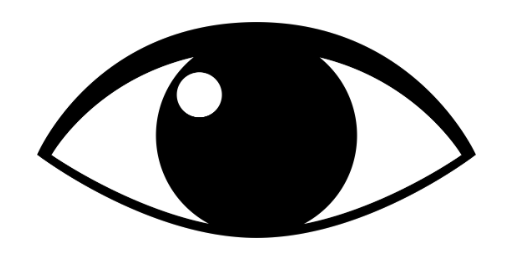 |
| 8 | % of direction errors | Number of direction errors divided by number of viable trials | 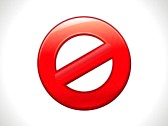 |
| 9 | SRT of corrective saccades | Interval between the target appearance and the corrective saccade onset | 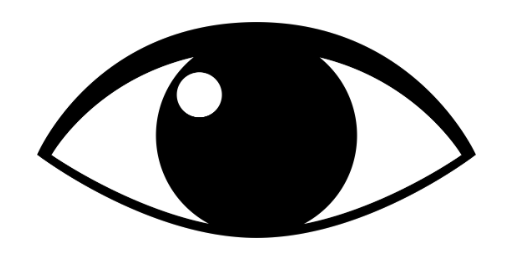 |
| 10 | % of corrected direction errors | Number of corrected direction errors divided by number of viable trials |  |
| 11 | % of anticipatory errors | Number of anticipatory errors divided by number of viable trials | 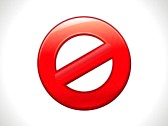 |
| 12 | % of trials with step saccades | Number of trials with step saccades divided by number of viable trials |  |
| 13 | Velocity | Speed of the saccade |  |
| 14 | Amplitude | The distance of the saccade |  |
| 15 | Angle between direct path and 1^st^ saccade | Angle between the line from the fixation point to the opposite of the target and the line from the 1^st^ saccade starting point to endpoint |  |

%: percent; SRT: saccadic reaction time; SD: standard deviation

: Accuracy; : Variability; : Main sequence;
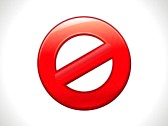
: Response inhibition; : other sensorimotor measures


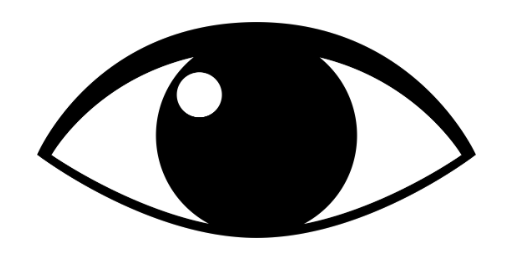


Table S3. Measurements of memory-guide saccades

| Index | Measurement | Description | Category |
| --- | --- | --- | --- |
| 1 | % of correct trials | Number of correct trials divided by number of viable trials |  |
| 2 | SRT of 1^st^ saccade of correct trials | Interval between FP disappearance and the first saccade onset in correct trials | 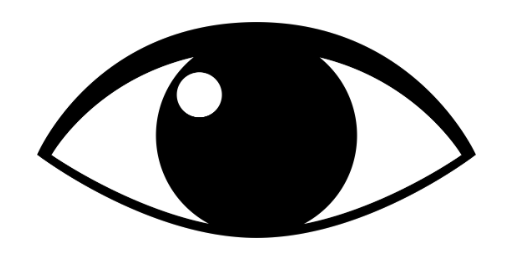 |
| 3 | SD of SRT of 1^st^ saccade for correct trials | Standard deviation of the SRT representing the amount of variation within an individual’s reaction time |  |
| 4 | Coefficient of variation of SRT of 1^st^ saccade of correct trials | Relative variability of the SRT within an individual’s reaction time |  |
| 5 | SRT of 2^nd^ saccade of correct trials | Saccadic reaction time to the second target | 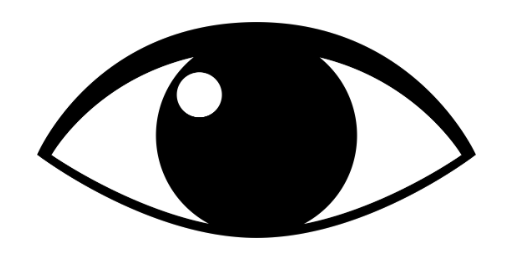 |
| 6 | SD of SRT of 2^nd^ saccade of correct trials | Standard deviation of the SRT representing the amount of variation within an individual’s reaction time |  |
| 7 | Coefficient of variation of SRT of 2^nd^ saccade of correct trials | Relative variability of the SRT within an individual’s reaction time |  |
| 8 | % of sequence errors | Number of sequence errors divided by number of viable trials |  |
| 9 | % of trials where they skipped the 1^st^ target and went to 2^nd^ only | Number of such trials divided by number of viable trials | 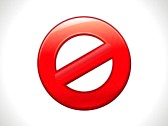 |
| 10 | % of trials where they went to the 1^st^ target only and stopped | Number of such trials divided by number of viable trials |  |
| 11 | % of trials that are sequence and timing errors | Number of such trials divided by number of viable trials |  |
| 12 | % of timing errors | Number of timing errors divided by number of viable trials | 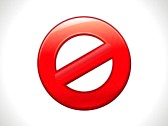 |
| 13 | % of false starts with sequence errors | Number of such trials divided by number of viable trials |  |
| 14 | % of false starts only | Number of such trials divided by number of viable trials | 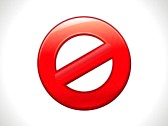 |
| 15 | % of all timing errors | Number of timing errors across all trials (those with other errors) divided by number of viable trials | 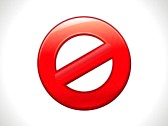 |
| 16 | % of all sequence | Number of sequence errors across all trials (those with other errors) divided by number of viable trials |  |
| 17 | % of trials with step saccades | Number of trials with step saccades divided by number of viable trials |  |
| 18 | Velocity of the 1^st^ saccade | Speed of the 1^st^ saccade |  |
| 19 | Amplitude of the 1^st^ saccade | The distance of the 1^st^ saccade |  |
| 20 | Velocity of the 2^nd^ saccade | Speed of the 2^nd^ saccade |  |
| 21 | Amplitude of the 2^nd^ saccade | The distance of the 2^nd^ saccade |  |
| 22 | Angle between direct path and 1^st^ saccade | Angle between the line from the fixation point to the target and the line from the 1^st^ saccade starting point to endpoint |  |
| 23 | Accuracy of the 1^st^ saccade | The distance (in degrees) the end of the first saccade is to the target |  |
| 24 | Accuracy of the final fixation to the 2^nd^ target | The distance (in degrees) the end of all saccades saccade is to the target |  |
| 25 | Accuracy of the closest fixation to T1 | The distance (in degrees) the end of the all additional corrective saccades is to the target |  |
| 26 | Path length accuracy (actual/optimal path length) | The length of the actual eye movement path divided by the length of the optimal path to both targets |  |

%: percent; SRT: saccadic reaction time; SD: standard deviation

: Accuracy; : Variability; : Main sequence; : other sensorimotor measures;
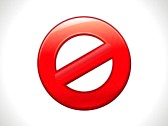
: Response inhibition; : Working memory


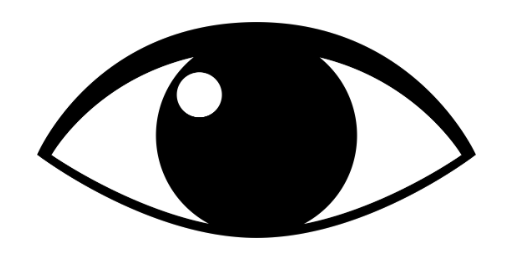


Table S4. Measurements of psychometric tests

| Test | Domain | Subtests | Age | Measurement |
| --- | --- | --- | --- | --- |
| NEPSY-II | Attention and executive functioning | Animal sorting | 7-16 | Animal sorting total correct scaled score |
|  |  |  |  | Animal sorting combined scaled score |
|  |  | Auditory attention and response set | 7-16 | Auditory attention (AA) combined scaled score |
|  |  |  |  | Response set (RS) combined scaled score |
|  |  |  |  | AA vs. RS contrast scaled score |
|  |  | Inhibition | 5-16 | Inhibition-naming completion time scaled score |
|  |  |  |  | Inhibition-naming combined scaled score |
|  |  |  |  | Inhibition-inhibition completion time scaled score |
|  |  |  |  | Inhibition-inhibition combined scaled score |
|  |  |  |  | Inhibition-switching completion time scaled score |
|  |  |  |  | Inhibition-switching combined scaled score |
|  |  |  |  | Inhibition total errors scaled score |
|  | Memory and learning | Memory of names | 5-16 | Memory for names total scaled score |
|  |  |  |  | Memory for names delayed total scaled score |
|  |  |  |  | Memory for names and memory for names delayed total scaled score |
|  | Visuospatial processing | Arrows | 5-16 | Arrows total score scaled score |
| WMTB-C | Working memory | Digit recall | 5-15 | Digit standard score |
|  |  | Block recall |  | Block standard score |
| WJ-III | Math ability | Quantitative concept | 5-75 | Standard Score |
| WRMT | Reading ability | Word identification | 5-75 | Word Identification standard score |

# Classifier Performance

Figure S1 shows the test accuracies of classifiers trained on the original training set (blue bars), and the average test accuracies of classifiers trained on resampled training sets (grey bars, with error bars showing the standard errors). One-way ANOVA test was used to compare these accuracies of different classifiers, and the Tukey’s honestly significant difference procedure was applied when multiple-comparison was involved. The significance level was set to be 0.05.

Classifiers trained on resampled training sets were more biased compared to those trained on the original training set since fewer unique training samples were included. So for majority of the classifiers, the average accuracies based on resampled training sets tended to be lower than the original ones, except some involving the psychometric tests, indicating a better generalizing ability of classifiers trained with psychometric data.

For classifiers trained on data of single assessment, the psychometric classifier yielded the best classification accuracy. Classifiers trained on age-corrected saccadic eye movement data performed better than those trained on the raw data for all of the three tasks. For pair-wise assessments, the accuracies on resampled training sets show slightly different results compared to the accuracies on the original training set. However, classifiers trained on combinations with psychometric tests data still performed the best (with no significant difference between combinations), followed by classifiers on AntiSac and natural viewing, and ProSac and natural viewing (no significant difference between these two). The best performance was achieved with the combination of data from four assessments (ProSac, AntiSac, Psychometric and Natural Viewing).

**Supplementary Figure 1.** Test accuracies of classifiers trained on the original training set (blue bars), and the average test accuracies of classifiers trained on resampled training set (grey bars, with error bars showing the standard errors). Dashed line shows the chance level (52.17%). **a)** Classifier performance on single assessment. **b)** Classifier performance on 4 assessments (age-corrected ProSac and AntiSac, Natural Viewing and Psychometric). **c)** Classifier performance on pair-wise assessments.

# Supplementary References

1. Chudley AE, Conry J, Cook JL, Loock C, Rosales T, LeBlanc N. Fetal alcohol spectrum disorder: Canadian guidelines for diagnosis. Canadian Medical Association Journal (2005) 172:S1–S21. doi: 10.1503/cmaj.1040302
2. Kowler E. Eye movements: The past 25years. Vision Res (2011) 51:1457–83. doi: 10.1016/j.visres.2010.12.014
3. Sparks DL. The brainstem control of saccadic eye movements. Nat Rev Neurosci (2002) 3:952–64. doi: 10.1038/nrn986
4. Platt ML, Ghazanfar AA. Primate neuroethology. Oxford University Press (2012). 201 p.
5. Land MF. Motion and vision: why animals move their eyes. J Comp Physiol A (1999) 185:341–52.
6. Yerram S, Glazman S, Bodis-Wollner I. Cortical control of saccades in Parkinson disease and essential tremor. J Neural Transm (2013) 120:145–56. doi: 10.1007/s00702-012-0870-3
7. Korkman M, Kirk U and Kemp S. NEPSY-II: A developmental neuropsychological assessment. San Antonio, TX, Harcourt Assessment; 2007.
8. Baddeley AD, Hitch G. Working memory. Psychology of learning and motivation (1974) 8:47–89.
